# Ion-to-Image, i2i, a Mass Spectrometry Imaging Data Analysis Platform for Continuous Ionization Techniques

**DOI:** 10.1021/acs.analchem.3c01615

**Published:** 2023-07-28

**Authors:** Johan Lillja, Kyle D. Duncan, Ingela Lanekoff

**Affiliations:** † Department of Chemistry − BMC, Uppsala University, Uppsala, 752 37, Sweden; ‡ Department of Chemistry, Vancouver Island University, Nanaimo, British Columbia V9R 5S5, Canada

## Abstract

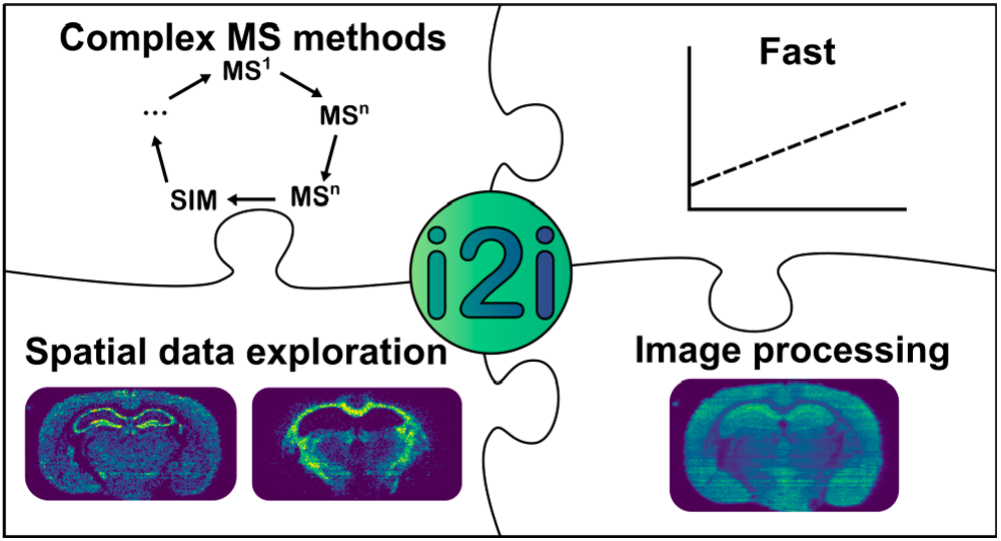

Mass spectrometry imaging (MSI) techniques generate data that reveal spatial distributions of molecules on a surface with high sensitivity and selectivity. However, processing large volumes of mass spectrometry data into useful ion images is not trivial. Furthermore, data from MSI techniques using continuous ionization sources where data are acquired in line scans require different data handling strategies compared to data collected from pulsed ionization sources where data are acquired in grids. In addition, for continuous ionization sources, the pixel dimensions are influenced by the mass spectrometer duty cycle, which, in turn, can be controlled by the automatic gain control (AGC) for each spectrum (pixel). Currently, there is a lack of data-handling software for MSI data generated with continuous ionization sources and AGC. Here, we present ion-to-image (i2i), which is a MATLAB-based application for MSI data acquired with continuous ionization sources, AGC, high resolution, and one or several scan filters. The source code and a compiled installer are available at https://github.com/LanekoffLab/i2i. The application includes both quantitative, targeted, and nontargeted data processing strategies and enables complex data sets to be processed in minutes. The i2i application has high flexibility for generating, processing, and exporting MSI data both from simple full scans and more complex scan functions interlacing MS^n^ and SIM scan data sets, and we anticipate that it will become a valuable addition to the existing MSI software toolbox.

## Introduction

Biological tissues are complex heterogeneous arrangements of various cell types and contain many substructures with unique chemical microenvironments and biological functions. Pioneering work in biological mass spectrometry imaging (MSI) introduced in the late 1990s and early 2000s enabled the visualization of molecular distributions in thin tissue sections without any chemical labeling or tagging.^1,2^ Recent advances in MSI techniques facilitates the simultaneous generation of hundreds to thousands of ion images showing molecular distributions of small and large molecules, including lipids, metabolites, proteins, and drugs.^3−8^ Compared to traditional methods, where the tissue is homogenized prior to analysis, the additional dimension of localization contributed by MSI enhances biological understanding by revealing the chemical microenvironments. Some recent examples utilizing the spatial distribution to study in situ tissue molecular aberrations include animal models for cancer,^9,10^ diabetes,^11,12^ and ischemic stroke.^13,14^

The most widely used MSI techniques include time-of-flight secondary ionization mass spectrometry (ToF-SIMS), matrix-assisted laser desorption/ionization (MALDI), and liquid-extraction MSI techniques such as desorption electrospray ionization (DESI) and nanospray desorption electrospray ionization (nano-DESI).^15−19^ Depending on the mechanism to introduce ions into the MS, MSI techniques can be classified as either pulsed or continuous ionization techniques. For example, the laser in MALDI and the primary ion beam in ToF-SIMS are operated in a pulsed mode to desorb and ionize analytes from specified regions in the tissue. By rastering the pulsed beam in a defined pattern across the tissue, the mass spectra acquired in MALDI and ToF-SIMS images are stored in grids with information on their origin and pixel sizes.^1^ Conversely, the events of desorption and ionization in nano-DESI and DESI are continuous along an axis, as the sample is moved under the probe to generate a line scan. Ion images are created by acquiring several line scans while the line is stepped in the perpendicular axis. For these continuous ionization techniques, the mass spectra are stored as an array of individual lines, usually as separate MS files instead of grids. Additionally, the pixel size in continuous ionization techniques is determined by the acquisition speed of the mass spectrometer, the operator-defined sample movement along the parallel axes, and the step between lines in the perpendicular axis. Thus, the use of a pulsed or continuous ionization technique for MSI dictates how the information for the spatially resolved MS spectra is stored.

Molecular annotation in both pulsed and continuous MSI techniques is fully dependent on the mass spectrometer’s mass accuracy and mass resolving power. The high mass accuracy, high mass resolving power, and high scan speed of the Orbitrap Fourier transform mass spectrometers (FTMS) make them attractive to use in combination with pulsed and continuous MSI techniques.^20−26^ In these systems, the automatic gain control (AGC) dynamically adjusts the ion injection time (IT) in each cycle to reach a set number of charges in the trap, minimizing space charge effects, and maximizing the dynamic range.^27,28^ For MSI applications, the AGC is sometimes turned off, however, this could negatively impact the dynamic range and mass accuracy in different tissue regions with varying cell densities due to differences in ion flux.^25−29^ Unfortunately, by using the AGC, the duty cycle is slightly different for each acquired mass spectrum, which complicates data processing. In particular, the number of spectra per acquired line in continuous ionization MSI and the size of each pixel along the axis of the image will vary. Therefore, data storage and image handling software tools for techniques utilizing AGC require customized strategies to accommodate the dynamic duty cycle and pixel size.

There is a vast amount of software available for data processing of MSI images in the .imzML format, which has a defined number of pixels. The deterministic number of pixels enables efficient data processing, and current software tools are able to perform quantification, annotation, and statistical analysis. Some examples of these software solutions include, but are not limited to, msIQuant, msiReader, Cardinal, and METASPACE.^30−34^ However, this predefined format is not suitable for continuous MSI techniques that leverage AGC for improved spectral quality from the mass spectrometer. The selection of software for this type of data is currently limited to MSIQuickView^35,36^ and PeakByPeak (Spectroswiss, Lausanne, Switzerland). Although MSIQuickView was top of the line when it was first presented, it is limited in its functionality and speed compared with today’s standards. Moreover, the modern PeakByPeak is a proprietary software suite that requires a purchased license.

In addition to continuous MSI techniques that use ACG, there is an increasing number of publications showing complex experimental setups with multiple scan filters. For example, with the continuous MSI technique nano-DESI, the literature includes several reports on complex MSI experiments with up to 92 interlaced MS^2^ scans and imaging in MS^4^ for increased selectivity.^37,38^ Along with the increased use of complex MS experiments in combination with MSI, there is a need for software tools that enable the data processing of complex MS experiments. However, neither MSIQuickView nor PeakByPeak can process combined complex scan functions that may include one or more ion activation event(s) (MS^n^ with any available activation method) for structural elucidation or selected ion monitoring window(s) for increased sensitivity. Furthermore, neither of these software solutions can perform nontargeted data analysis of the large number of detected peaks in an experiment, which leaves most of the generated data unexplored. To make it possible to generate and process images acquired with AGC and with complex combinations of scans in both targeted and nontargeted modes, we have developed our own software solutions.

Here, we describe a new analysis software, ion-to-image (i2i), which is capable of handling the dynamic pixel sizes acquired with continuous MSI techniques and AGC. The user-friendly i2i application is written in MATLAB and includes ion image generation for both targeted and nontargeted analysis, image scaling, region-of-interest analysis (ROI), normalization and quantification, and free selection of scan filters for multiscan imaging experiments. We foresee that i2i will be a very useful tool for the growing imaging community using continuous MSI techniques and AGC generated data.

## Materials and Methods

### Development

A standard workstation computer with an Intel Xeon E3-1245 V6 and 16 GB of RAM with an NVMe SSD hard drive was used throughout the development and testing. The program was developed in MATLAB 2022a (MathWorks Inc., Natick, MA, U.S.A.) with the Bioinformatics Toolbox, Parallel Computing Toolbox, Image Processing Toolbox, and MATLAB App Designer. The colormap viridis and copyUIaxes functions were downloaded from MATLAB file exchange.^39,40^

### Test Data Set

The testing data set for this study was generated with pneumatically assisted (PA) nano-DESI MSI of a 10 μm thick mouse brain section purchased from Creative Biolabs (Shirley, NY, U.S.A.). Details of the nano-DESI experimental setup are described elsewhere.^36^ Briefly, 150 μm o.d. 50 μm i.d. fused silica capillaries were assembled as the nano-DESI probe, and the ion images were acquired using a 75 μm step size between lines to oversample the tissue section 2 times.^41^ The XYZ stages were controlled with custom LABView software, where the sample was moved along the *x*-axis at 20 μm·s^–1^.^36^ The solvent was 9:1 HPLC-MS grade acetonitrile (Merck, Darmstadt, Germany) and methanol (Merck, Darmstadt, Germany) with 10 ppm of ^107^Ag^+^ (Trace Sciences International, Richmond Hill, ON, Canada) with 0.5 μM PC 25:0 (Merck, Darmstadt, Germany) as the internal standard and was supplied at a flow rate of 0.5 μL·min^–1^ with a syringe pump (KD Scientific Inc., Holliston, MA, U.S.A.). An Orbitrap Velos Pro (Thermo Fisher, Bremen, Germany) was used with both FTMS and MS^n^ scans interlaced. A total of 92 line scans were collected with a total data set file size of 3.3 GB. Each line scan in the data set was converted to .mzML and centroided using ProteoWizard MS Convert, resulting in an average .mzML file size of 14 MB.^42^

## Results and Discussion

**Figure 1:**
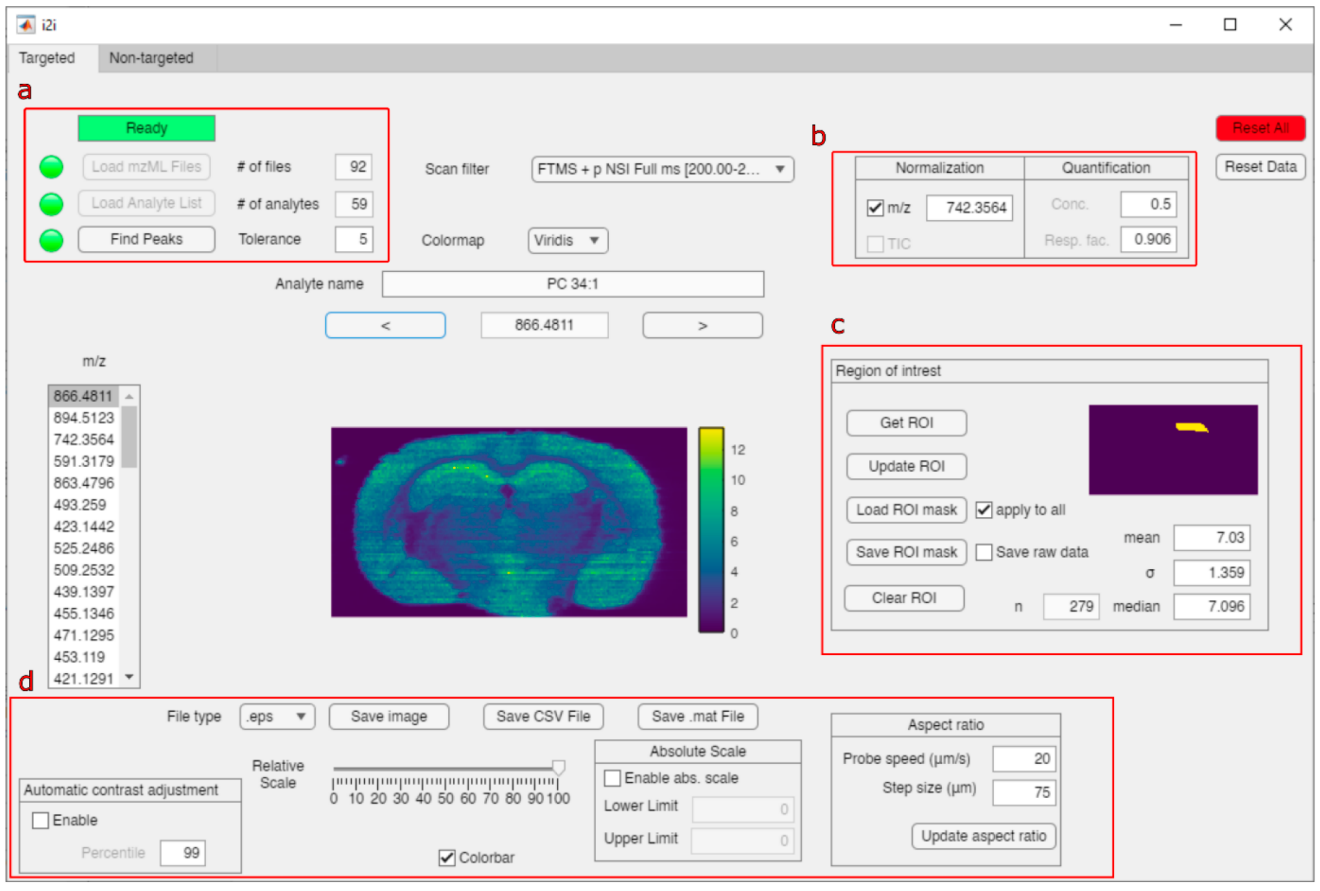
GUI of the main window of the i2i application showing the targeted mode with (a) the data input and data extraction module, (b) the normalization and quantification module, (c) the region-of-interest (ROI) module, and (d) the image scaling and export module.

Easy and accessible ion image generation, processing, and analysis by i2i are facilitated in a graphical user interface (GUI) written in the MATLAB app developer. The i2i application offers individual tools for data and feature input, image viewing, image normalization, image scaling, one-point quantification, and region-of-interest (ROI) analysis (Figure 1). The operator can select to visualize ion images with different colormaps,^39^ tune the image brightness based on intensity or relative abundance, and remove hot pixels (i.e., pixels with extreme outlying intensities) with the automatic contrast adjustment tool, which sets the color scale maximum to the user defined *n*th percentile of the intensities. Additionally, the i2i software enables customized features based on experimental parameters. These include quantification of selected analytes by using internal standards of known concentrations, setting the image aspect ratio by inputting the sample speed along the axis and the step size in the perpendicular axis, and selecting scan filters used for data acquisition for image generation. Generated images can be exported as both images (as .eps, .png, and .tiff) or as matrices (as .mat and .csv) for subsequent data analysis. Thus, the i2i application offers high flexibility in image generation, processing, and analysis of both simple and complex MSI workflows, including multiple selected ion monitoring scans, MS^n^, and polarity switching.

**Figure 2:**
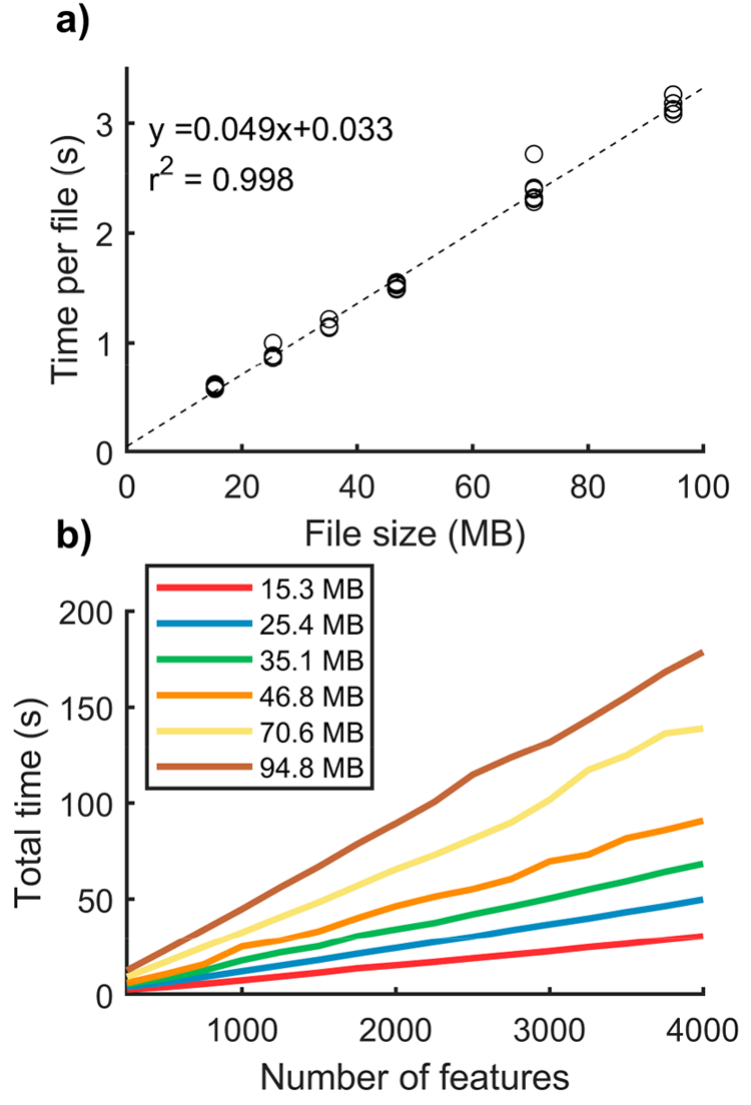
Performance metrics for the i2i functions to read .mzML files and extract targeted features. (a) The time needed to read a file as a function of file size. The regression line shows that the file loading time increases with 49 ms per MB. (b) Performance metrics for extraction of targeted *m*/*z* features. The *y*-axis shows the time for extracting a number of features (*x*-axis) from data files of different sizes. The file sizes vary between 15.3 MB (red) to 94.8 MB (brown).

For efficient loading and processing of data, all raw files are converted to the open file format .mzML as centroids. Using centroided data in place of continuous data reduces the file size by a factor of 3 in our test data set, making further data processing less computationally demanding and manageable in real-time on common workstations. However, since the data is reduced, manual verification of the detected peak in the raw data is encouraged. To read the vendor-neutral .mzML files into i2i, a .mzML file reader function was written using the built-in *readstruct* function in MATLAB. The .mzML reader rapidly extracts all necessary parameters from files for downstream data processing, including the mass spectrum, scan filter, total ion current (TIC), and time for each scan event. File loading from the 3.3 GB testing data set was completed in 92 s, corresponding to ∼1 s per .mzML file (∼14 MB), and extraction of *m*/*z* features for targeted analysis was completed in milliseconds. Figure 2a shows the performance of the file reader function and that file loading time scales linearly with the file size (49 ms per MB). In the i2i application, the parallel computing toolbox is utilized to increase the throughput by loading files in parallel. Following file loading and ion image generation, the user can process the data with either a targeted or nontargeted workflow, which is discussed in detail below.

Although AGC in Orbitrap MS systems improves spectra quality, the resulting dynamic pixel sizes complicate ion image generation. In continuous ionization techniques where MSI data is acquired in lines, the dynamic pixel sizes may result in each line having different numbers of pixels with dynamic pixel widths. Consequently, ion images will be distorted if all pixels are assumed to be equal size. This is especially visible at the beginning of each line where the differences accumulate and can be seen as irregular tissue features and edge (Figure S1a). Here, we mitigate the effects of dynamic pixel sizes by constructing a virtual time axis from *t*_0_ to *t*_max_, where *t*_max_ is the longest time in all line scans.^35,36^ The actual intensities in each position are then interpolated to the closest time using the *interp1* function in MATLAB. Specifically, we implement closest value interpolation to obtain the truest representation of the data despite the possibility of interpolating using a linear gradient or spline interpolation. This interpolation efficiently aligns the pixels and removes any distortion caused by the varying pixel sizes by AGC (Figure S1b). Thus, we rapidly and robustly align ion images with dynamic pixels while keeping the valuable AGC feature of the Orbitrap mass analyzer.

### Targeted Data Analysis

Targeted data analysis in i2i is the default option where known *m*/*z* values are extracted to construct ion images from a data set. The user lists the targeted *m*/*z* values in a spreadsheet, with the monoisotopic masses in the first column and the molecular annotation (optional) in the second column (Figure 1a, “Load Analyte List”). If the annotation is listed, it is displayed above the ion image in the i2i GUI, if not, the GUI shows “feature not annotated”. Following, i2i extracts the features corresponding to the *m*/*z* values in the list from each spectrum. To increase the speed of the algorithm, a difference matrix is calculated between the *m*/*z* in each spectrum and each *m*/*z* value in the target list instead of calculating differences individually for each data point. The calculated mass error in the difference matrix is compared to the user-defined parts per million threshold, and the algorithm returns the *m*/*z* and intensity for the feature with the lowest ppm error. The computational speed is also increased by reading several .mzML files in parallel. In Figure 2b, the performance of this function is shown when extracting 250 to 4000 random *m*/*z* features from files of varying sizes. The function scales linearly with both file size and the number of features, where the time per feature ranges from 8 ms for a 15 MB file to 45 ms per feature for a 95 MB file. It should be noted that the absolute file loading and feature extraction times depend on the computer processor, hard drive, and RAM available. However, the linear scaling and short execution times demonstrate that the targeted i2i file loading and feature detection algorithms are suitable for most computers.

In addition to generating ion images, options for signal normalization are imperative in MSI data workflows. A common method employed by the MSI community involves normalizing the intensity of a selected *m*/*z* in each spectrum to the total ion current (TIC) in that spectrum. TIC normalization is a nonspecific normalizing strategy that mainly accounts for overall deviations during the ionization event and that does not account for the different ionization of individual analytes in a given matrix.^43^ In the continuous ionization MSI technique nano-DESI, the events of desorption and ionization are separated, enabling the simultaneous ionization of analytes with an internal standard doped into the nano-DESI solvent.^43,44^ With the inclusion of an appropriate internal standard, the analyte signal can be normalized to the signal of the internal standard for mitigation of matrix effects and one-point quantification.^20,43,45^ The i2i application enables easy selection for normalization to the TIC or any selected *m*/*z* (Figure 1b). Additionally, the operator can input the concentration of an included internal standard and its analyte response factor, which immediately updates the pixel values to show a quantitative ion image. The quantitative ion images generated in i2i enable reliable visualization of the targeted analytes throughout the tissue section.

Features in i2i enable in-depth data analysis through ROI analysis (Figure 1c). The ROI analysis in i2i enables intensities or detected concentrations of analytes to be compared across different morphological substructures in a tissue section. The user selects the “Get ROI” button and manually draws the desired ROI directly on an ion image. Following this, the data from the ROI is presented as descriptive statistics on the pixel values in the area. In addition to obtaining the direct values, the user can choose to save the ROI data for further processing and comparison. The user can then apply the same ROI across all desired mass channels for the rapid generation of spatially resolved and averaged intensity data or save the ROI masks to reload for future sessions or evaluation in a different software.

### Nontargeted Analysis

**Figure 3:**
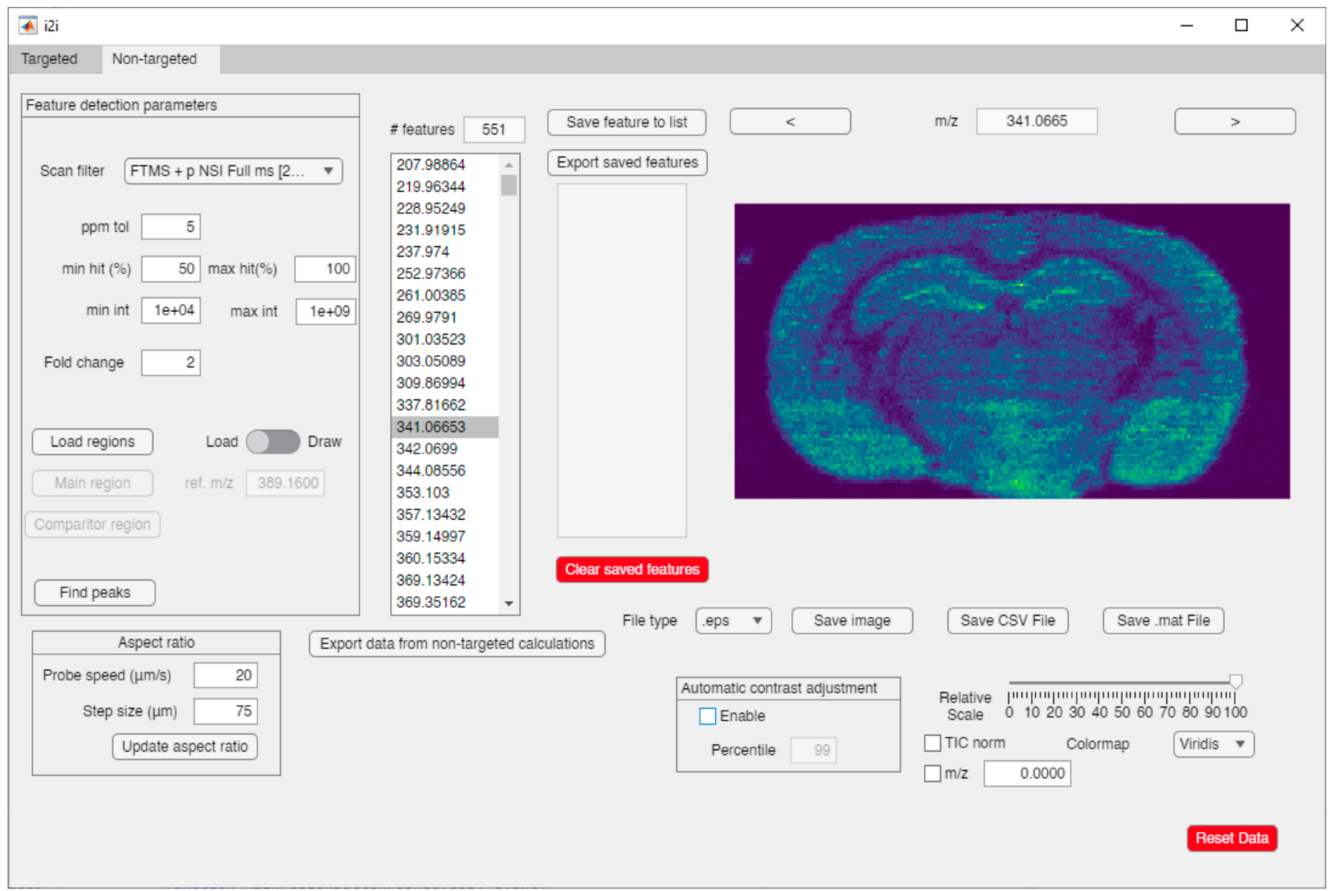
GUI of nontargeted i2i showing the feature detection parameters including ROI, basic image normalization and adjustment strategies, and a list of detected feature groups that the user can browse through manually or save.

The targeted workflow in i2i enables the rapid visualization of the distribution of selected analytes in tissue but is limited to user-selected *m*/*z* values. Nontargeted data analysis unlocks the full potential of MSI data to provide unique opportunities for data exploration and hypothesis generation. The nontargeted i2i GUI utilizes the same processing and analysis of ion images as the targeted i2i, without using a user-defined target list of *m*/*z* values (Figure 3). The user simply selects one *m*/*z* value for generating a reference ion image to start data processing. Following, the user can use ROI analysis in two different ways: either to draw an ROI on the reference ion image to obtain full information on all detected features in the defined region or to draw two different ROIs to obtain disparate features that are either accumulated or depleted between the two ROIs. For example, the two regions could be comparing data from tissue versus data on glass to filter background peaks. Another option is to select two different ROIs on the tissue to compare detected *m*/*z* features in distinct morphological regions. As an alternative to loading a reference ion image, the user can directly load previously saved ROIs from the ROI toolbox. After the nontargeted search, the algorithm returns information on unique or upregulated *m*/*z* features, including *m*/*z* value and intensity, which can be saved as a .csv for further processing.

A key challenge for the nontargeted analysis of MSI data is spectral alignment of detected *m*/*z* values, also referred to as features. Due to instrumental operations, the exact value for the centroid *m*/*z* deviates slightly between spectra (Figure S2). Thus, it is essential to properly align the features. Binning is an established and algorithmically simple technique. However, the size of each bin is crucial to retain mass accuracy while not artificially generating nonexistent features.^46^ Furthermore, in Orbitrap FTMS data resolution is proportional to the inverse square root of *m*/*z*, which makes fixed bin widths inappropriate.^47^ To overcome these limitations, the i2i algorithm is designed to group all of the detected *m*/*z* features into feature groups within a given tolerance in ppm. This will provide feature groups corresponding to the data, without generating nonexistent features or splitting existing features. In i2i, this is done efficiently and accurately, as exemplified by the i2i algorithm grouping 95% of 1804 features within 0–3.2 ppm in seconds (Figures S3 and S4). Thus, the nontargeted i2i provides a convenient and simple way to rapidly explore the entire image data set.

**Figure 4:**
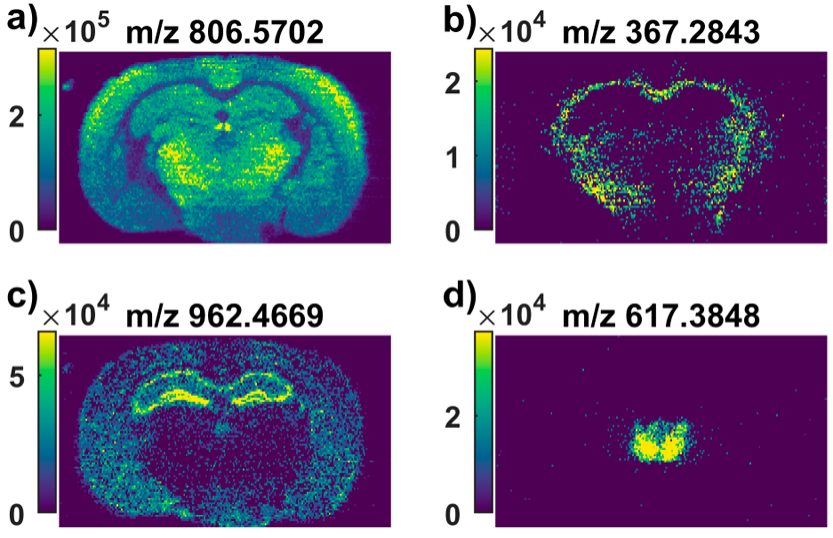
Examples of ion images generated by the nontargeted algorithm when comparing different regions at different intensity thresholds and detection frequency settings: (a) Comparing the whole tissue region to glass with intensities 1E5–1E9 with detection frequencies between 50 and 100%; (b) Comparing the whole tissue region to glass with intensities 1E4–1E9 with detection frequencies between 10 and 30%; (c) Comparing the hippocampus to the thalamus with intensities between 1E4–1E9 and detection frequencies between 10 and 100%; (d) Comparing the thalamus to the hippocampus with intensities between 1E4–1E9 and detection frequencies 10–100%.

An important component of the nontargeted i2i is that the user can filter the nontargeted list of feature groups based on intensity, detection frequency in the ROI, and fold change. By tweaking these parameters, it is possible to select images based on location, intensity, and detection frequency (Figure S5). This is especially important for quickly identifying ion images of feature groups with low overall intensity but specific localization or to filter away feature groups that are sparsely detected. For example, when comparing the entire tissue section to the surrounding glass slide with a detection frequency set between 50 and 80% and the intensity threshold for peaks above 1 × 10^5^, ion images of abundant and homogeneous feature groups are generated (Figure 4a). If the detection frequency is instead lowered to between 10 and 30% and the intensity threshold is lowered to 1 × 10^4^, ion images for feature groups with a low intensity that are only represented in part of the tissue are generated (Figure 4b). Alternatively, two different regions on the tissue can be compared to find feature groups with a unique localization in one of the regions. This is represented by the clear differences in lipid localizations revealed between the thalamus and hippocampus in our test data set (Figure 4c,d). Thus, both nontargeted visualization and nontargeted data mining are possible with i2i, making it a useful tool in data exploration and hypothesis generation.

## Conclusion

We have presented the i2i application, which provides a simple tool for the generation and data processing of ion images acquired by continuous ionization sources using AGC. By utilizing centroided .mzML data and an efficient MATLAB code, ion images can be rapidly generated, processed, and exported in both targeted and untargeted modes. The i2i GUI enables the user to tailor the ion images based on specific experimental parameters, select the desired scan filter, scale to observed signal intensities, set the true aspect ratio, and normalize to the TIC or any *m*/*z* value, such as an internal standard. The application further enables quantitative workflows, where the concentration and response factor of an internal standard can be used to generate quantitative ion images and ROI data. Finally, by the efficient calculation of feature groups, the nontargeted workflows of i2i allow the user to explore the entire data set and compare data without restrictions. Overall, i2i fills the gap for image data generation, processing, and exploration for MSI techniques relying on continuous ionization sources and high quality spectra acquired by using AGC.

## Data Availability

The source code and a compiled version of the installer is available at https://github.com/LanekoffLab/i2i.

## Supporting Information Available

Additional data, performance metrics, discussion and description of the algorithms (PDF)
