# Supplementary material for: Ion-to-Image, i2i, a Mass Spectrometry Imaging Data Analysis Platform for Continuous Ionization Techniques
Source: Anal Chem. 2023 Jul 28;95(31):11589–95. doi: 10.1021/acs.analchem.3c01615 (PMC10413325; doi:10.1021/acs.analchem.3c01615)
Supplement: Supplementary file 1 — ac3c01615_si_001.pdf [file ac3c01615_si_001.pdf]

Supporting information

Ion-to-image, i2i, a new mass spectrometry imaging data analysis platform for continuous ionization techniques

Johan Lillja[1], Kyle D. Duncan[1,2], Ingela Lanekoff[1*]

[1]Department of Chemistry – BMC, Uppsala University, Uppsala, 752 37, Sweden
[2] Department of Chemistry, Vancouver Island University, Nanaimo, BC V9R 5S5, Canada

Corresponding author:

Prof. Ingela Lanekoff

Ingela.Lanekoff@kemi.uu.se

Dept. of Chemistry-BMC (576)

Uppsala University

751 23 Uppsala

Sweden

Table of Contents

Figure S1 Time alignment example 1

Figure S2 Centroided peak data compared to continuous data 4

Figure S3 Histogram of mass errors in multiple feature groups 4

Figure S4 Feature grouping time as a function of ROI size 4

Figure S5 Characterization of parameters used for non-targeted searches 4

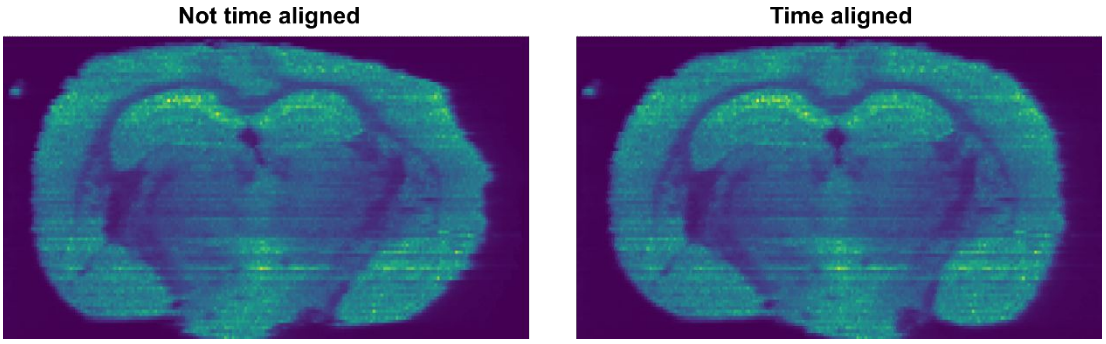

Figure S1. Ion images of PC 36:1 from a mouse brain tissue section with and without time alignment. The dataset was acquired by moving the sample along the x-axis from left to right at a speed of 20 µm/sec, and stepping in 75 µm increments along the y-axis from top to bottom. There is clear distortion in the rightmost edge of the tissue for the not time-aligned image compared to the time aligned due to scan-to-scan differences from the AGC.

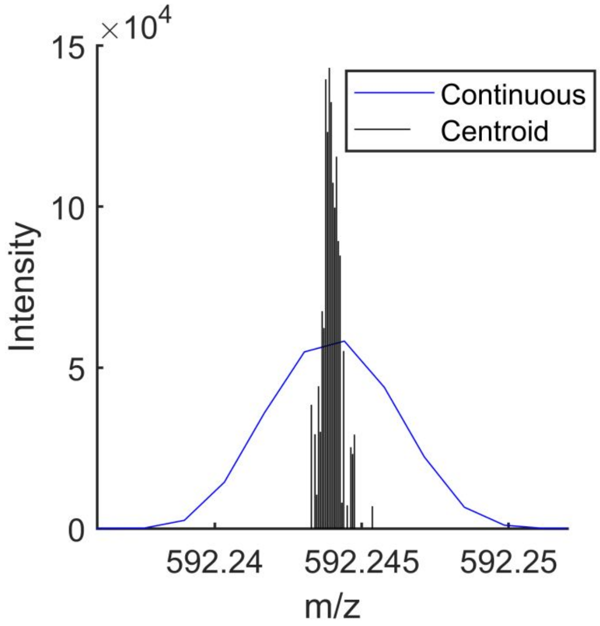

Figure S2. Continuous verses centroid data, where the averaged continuous data trace is shown in blue and the centroid peaks from from the .mzML file in black.

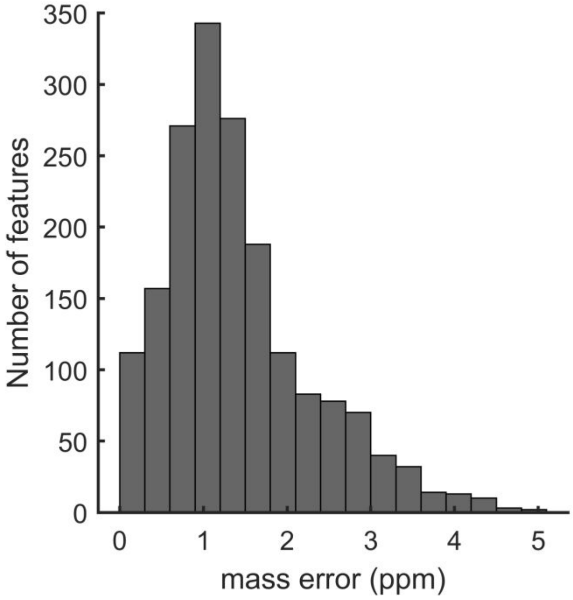

Figure S3. A histogram showing the average mass error within feature groups identified by the i2i non-targeted algorithm (n=1804, threshold set to 5 ppm). The 95th percentile was calculated to be 3.15 ppm.

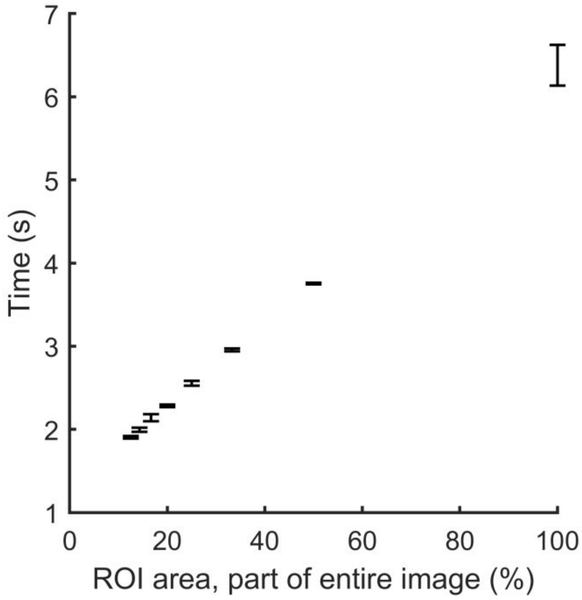

Figure S4. Processing time needed to identify feature groups originating from differently sized ROI areas from the imaging test data set. 100% represents the entire image, including glass, and 50% represents half of the entire image. The minimum detection frequency was set to 10 % and the minimum intensity to 5E3.

Characterization of detection frequency and minimum intensity threshold in the non-targeted algorithm

The effect of the minimum intensity and detection frequency parameters was quantified by calculating the correlation coefficient (r^2) of all detected feature groups to the ion image of PC 34:1, which shows a homogenous distribution. Two different cases were tested, either by comparing the whole tissue region to a region of glass (figure S5 a-b), or a small part of the white matter to a region of glass (figure S5 c-d). The results were divided into two groups; below and above an r^2 value of 0.15 as a cutoff between real features and noise. Feature groups found in the whole tissue section are shown in figure S5a-b and for the white matter subregion in figure S5c-d. The number of feature groups both above and below 0.15 r^2 increases as the intensity threshold is lowered. When the detection frequency is increased, the detected number of features with an r^2>0.15 decreases non-linearly when comparing the ROI of the entire tissue section to the ROI from part of the glass (Figure S5 a-b). The nonlinear effect was, however, not present when a small region of white matter was compared to glass since this area is relatively more homogeneous (Figure S5 c-d). Overall, this shows that the detection frequency can be used to efficiently filter away random to low abundant feature groups in the selected ROI.

The intensity threshold and detection frequency are therefore important to balance to minimize the number of noise features found from the non-targeted search, and will to some extent, have to be determined empirically depending on the experimental parameters used (MS, data quality, background signals) and the properties of the tissue section (heterogeneity of the selected region).

Peak grouping algorithm

The non-targeted algorithm is based on a peak grouper that defines unique *m/z* values in a subset of spectra. It does this by stacking all spectra in a defined subset in a vector and sorts them based on mass to charge value. The first element *i* is now compared to the element *i+1* for the mass accuracy tolerance, if the mass difference for the element *i* is above the tolerance it is saved as a unique value, if the mass difference for the element *i* is within the tolerance a weighted average m/z value is calculated based on intensity which is used for comparisons in the next iteration of the loop. The weighted average is updated at each iteration until the tolerance is above the user defined threshold. This process is iterated until all elements have been assigned to a feature group.

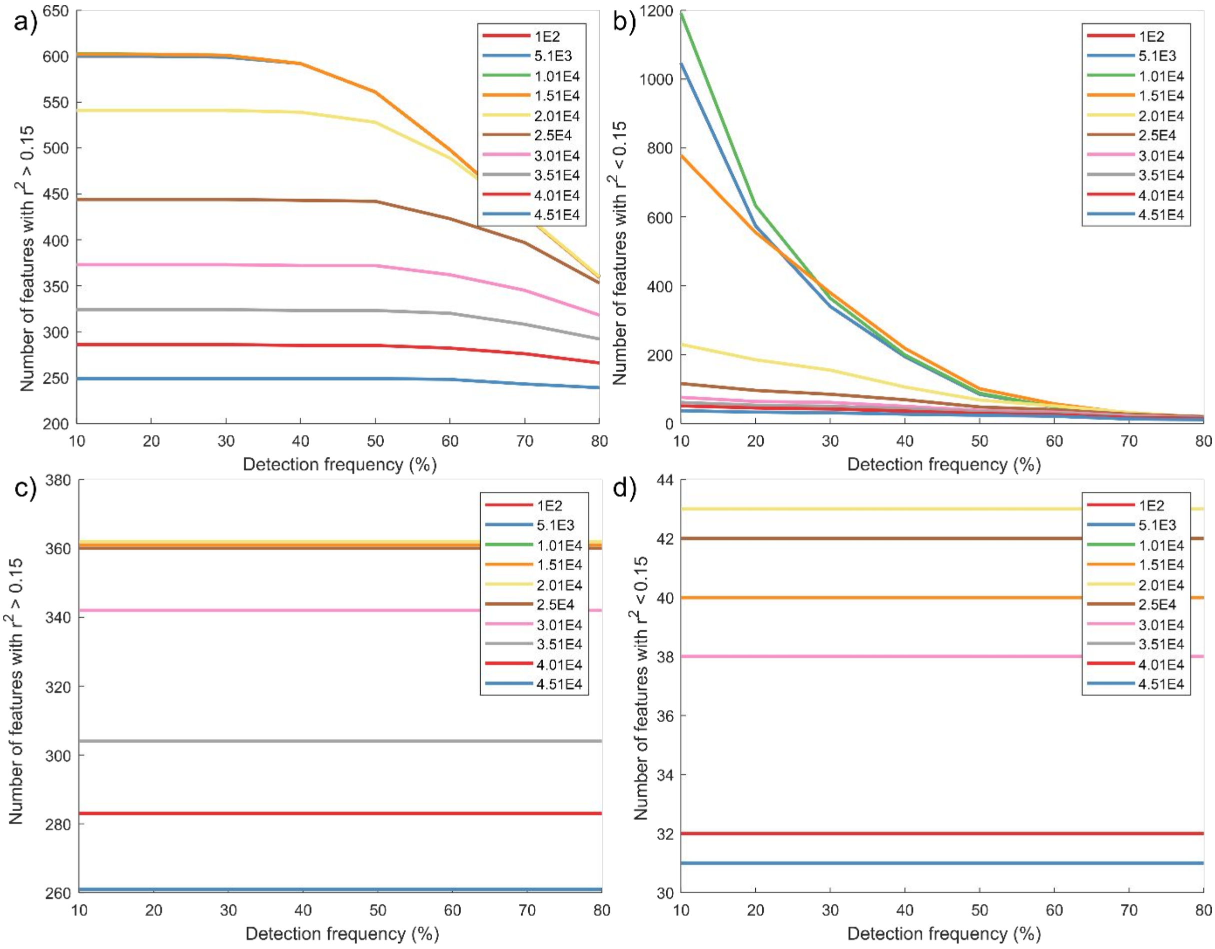

Figure S5: Correlation metrics compared to PC 34:1 for the non-targeted features found using different levels of detection frequency and 10 different intensity thresholds (shown in different colors) in different ROIs compared to glass. (a) The number of strongly correlating features ($r^2>0.15$) from the whole tissue section relative to glass (b) number of features with a weaker correlation ($r^2<0.15$) in the whole tissue section relative to glass (c) number of features with a strong correlation ($r^2>0.15$) in a small area of the white matter (d) number of features with a weak correlation in a small area of the white matter.
